# Primary cutaneous anaplastic large cell lymphoma: Clinical response with low dose brentuximab vedotin on a patient receiving hemodialysis

**DOI:** 10.1016/j.jdcr.2025.04.004

**Published:** 2025-04-17

**Authors:** Claire Fason, Stephen Tyring, Auris O. Huen

**Affiliations:** a Department of Dermatology, McGovern Medical School at UT Health Houston, Houston, Texas; b Department of Dermatology, The University of Texas MD Anderson Cancer Center, Houston, Texas

**Keywords:** anaplastic large cell lymphoma, brentuximab vedotin, CD30 lymphoproliferative disorder, cutaneous T-cell lymphoma, hemodialysis, renal dysfunction

## Introduction

Primary cutaneous anaplastic large cell lymphoma (PC ALCL) is a rare type of CD30^+^ lymphoproliferative disorder. Presentation is typically a single ulcerated nodule or a localized group of nodules, however rarely, PC ALCL presents with multiple nodules in various locations. Extracutaneous involvement occurs in 10% to 15% of patients and typically involves regional lymph nodes.^1^ Although some cases regress spontaneously, extensive cases often require therapy. Treatments of choice for PC ALCL currently consist of surgical excision, radiation, methotrexate, or bexarotene.^1^^,^^2^ Recently, brentuximab vedotin (BV), an anti-CD30 monoclonal antibody conjugate to monomethyl auristatin E, has been trialed as an effective systemic therapy for severe disease.^3^^,^^4^ The pharmacokinetics and safety profile of BV in patients with renal impairment has been investigated, however there is little data regarding the efficacy and safety in patients undergoing hemodialysis.^5^ To our knowledge, there are only 3 case reports demonstrating BVs efficacy in patients with Hodgkin lymphoma and anaplastic lymphoma kinase (ALK) negative systemic ALCL undergoing hemodialysis for end-stage renal disease (ESRD).^6^, ^7^, ^8^ We present an unusual case of widespread PC ALCL that has been successfully managed with low dose BV in a patient with diabetes and ESRD requiring hemodialysis who did not respond to radiation or bexarotene.

## Case report

A 77-year-old man with a significant medical history of diabetes, prostate cancer, and ESRD on hemodialysis presented to our clinic with approximately 300 papules and nodules on his trunk and extremities. He stated the lesions come and go, frequently ulcerate or develop into tumors that do not resolve, bleed spontaneously and are very bothersome. Biopsy at an outside clinic revealed CD30 lymphoproliferative disorder. Treatment with excision, narrowband UV-B phototherapy, bexarotene 300 to 600 mg were attempted, however none of these treatments successfully managed his disease. This led him to be referred to our clinic.

Upon presentation to our clinic, a repeat biopsy was done to confirm the diagnosis. Biopsy showed a proliferation of intermediately sized atypical mononuclear cells primarily involving the dermis. Upon immunohistological staining, the cells were found to be positive for CD30 and negative for CD2, CD3, CD7, CD8, and ALK. There were a few rare subsets of cells that stained positive for CD4 and CD5. The tumor was predominantly T-cell receptor (TCR) alpha-beta in phenotype (TCR beta F1 antibody directed against the β chain of the alpha/beta TCR was weakly positive), whereas TCR gamma antibody labeled a few scattered cells.

**Fig 1:**
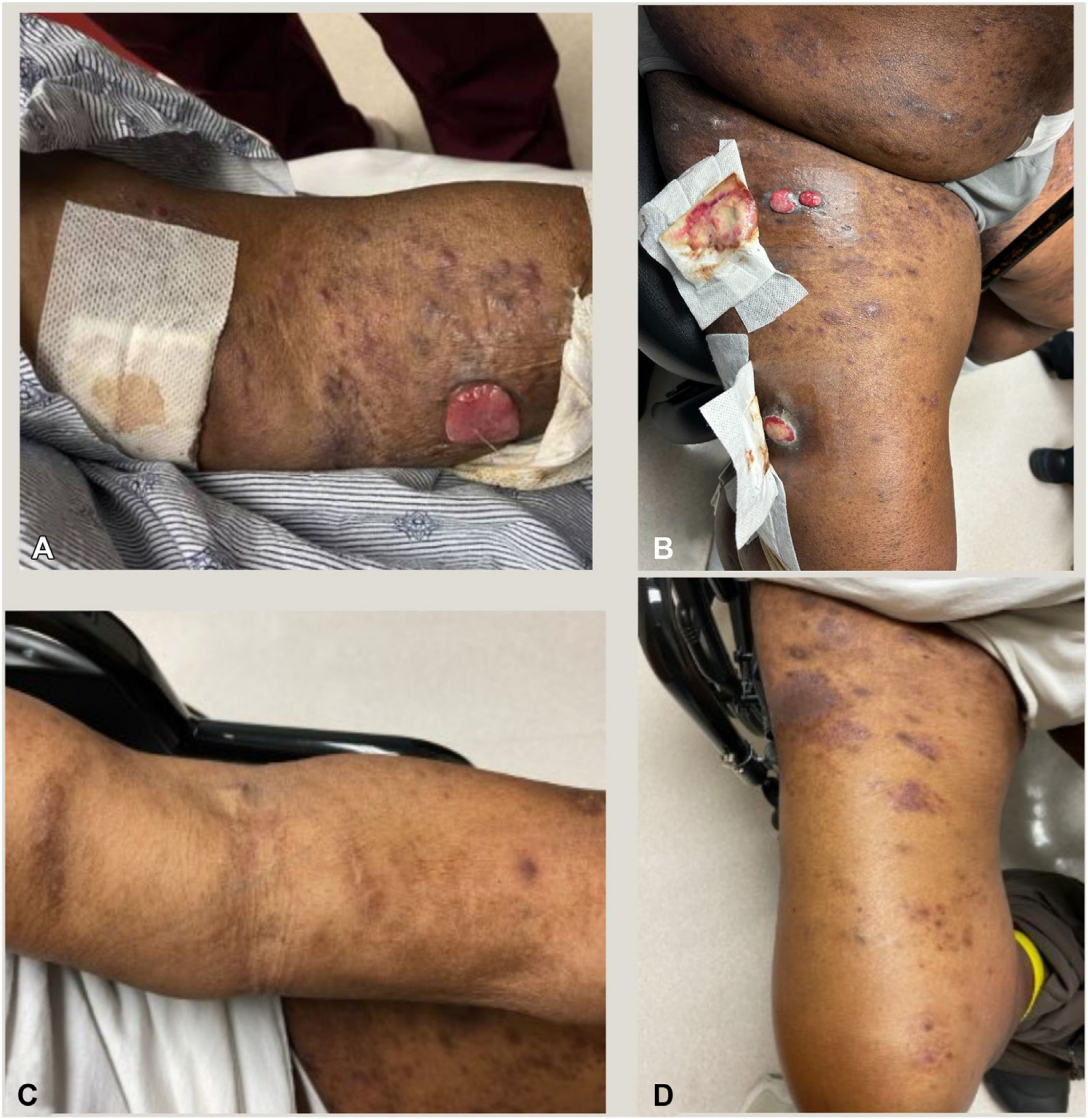
Clinical photographs of the patient before starting brentuximab vedotin (**A,** left arm and **B,** right thigh) and after treatment (**C,** left arm and **D,** right thigh).

Given the patient’s clinical presentation and the results of the biopsy, the final diagnosis was consistent with PC ALCL. Positron emission tomography or computed tomography showed multiple fluorodeoxyglucose (FDG) avid cutaneous lesions, but only a few reactive lymph nodes, not concerning for systemic disease. The patient underwent 600 cGy of radiation in 2 fractions, which transiently helped some lesions, however the lesions continued to recur; therefore, further doses of radiation therapy were deemed futile. Because of the patient’s ESRD, infusions of BV at a lower dose of 0.9 mg/kg every 4 weeks were initiated. Considerable skin improvement was seen after cycle 1, and after 3 cycles the patient achieved a complete response, so the decision was made to discontinue monthly infusions (see Fig 1).

Two months after his last infusion, the patient returned to the clinic with new lesions. Upon examination, there were >10 ulcerated tumors. BV infusions were restarted at 0.9 mg/kg every 4 weeks. A significant improvement of the lesions was noted, however, the patient reported worsening neuropathy in his right foot; therefore, further cycles of brentuximab were held. Because of recurrence of lesions soon after discontinuation, the patient required intermittent courses of higher-dose BV at 1.2 mg/kg administered intravenously every 3 weeks for the next 9 months with disease control. Neuropathy remains mild and tolerable.

## Discussion

First-line treatments for PC ALCL consist of methotrexate, radiotherapy, and surgical excision. For patients with liver disease, kidney disease, or if the disease is refractory to methotrexate therapy, bexarotene is routinely used.^2^^,^^3^ In our patient, methotrexate was not an appropriate option due to ESRD. His disease was not appropriately managed with radiotherapy or bexarotene. Surgical intervention is not indicated given the considerable number of lesions. According to an international, multicenter, phase 2 trial, 1.8 mg/kg of BV achieved better responses than both oral methotrexate and oral bexarotene.^4^

While the pharmacokinetics of BV use in patients with renal impairment have been investigated, there is limited data and recommendations regarding its use in patients with ESRD requiring hemodialysis. For those with renal impairment the current recommended dose is 1.2 mg/kg.^5^ Of the 3 case reports using BV in patients requiring hemodialysis, 2 detail management of classical Hodgkin lymphoma, whereas the third discusses using BV in a case of ALK negative systemic ALCL. For both cases of Hodgkin lymphoma, BV was dosed at 1.8 mg/kg, whereas 1.2 mg/kg was used for the ALK negative systemic ALCL.^6^, ^7^, ^8^

For our patient, a lower dose of 0.9 mg/kg was selected due to his comorbidities of renal failure on hemodialysis with uncertainty of drug clearance and diabetes, which may affect his risk of developing neuropathy. Initially, our patient had an excellent response to the lower dose of BV; however, he did report mild lower extremity neuropathy after 5 total cycles. It is unclear if the neuropathy was an adverse effect of BV, as the patient also had comorbid diabetes, which commonly causes peripheral neuropathy. Due to the significant disease improvement and the onset of neuropathy, the infusions of BV were stopped. Eventually, the patient required intermittent treatment with BV at higher doses for disease control. To our knowledge, it is the first reported case of BV use in a patient with PC ALCL with comorbid diabetes and ESRD, and additionally, a lower dose (0.9 mg/kg) of BV was trialed initially and produced a positive response. This case demonstrates the efficacy and tolerability of low dose BV in patients with PC ALCL and significant treatment limiting comorbidities, therefore indicating that BV is a viable treatment option for patients with PC ALCL whose disease has been refractory to other therapies, but response may not be durable after stopping treatment.

## Conflicts of interest

None disclosed.
